# Editorial: Emerging frontiers of microbial functions in sustainable agriculture

**DOI:** 10.3389/fmicb.2022.1128267

**Published:** 2023-01-11

**Authors:** Manoj Kumar Solanki, Asit Mandal, Flavio Henrique Vasconcelos De Medeiros, Mukesh Kumar Awasthi

**Affiliations:** ^1^Plant Cytogenetics and Molecular Biology Group, Faculty of Natural Sciences, Institute of Biology, Biotechnology and Environmental Protection, University of Silesia, Katowice, Poland; ^2^Indian Institute of Soil Science, Indian Council of Agricultural Research, Bhopal, India; ^3^Plant Pathology Department, Universidade Federal de Lavras, Lavras, Brazil; ^4^College of Natural Resources and Environment, Northwest A&F University, Xianyang, China

**Keywords:** plant microbiome, microbial symbiosis, mineral solubilizers, composting, fruit microbiome

## Introduction

Climate change scenarios have significantly impacted food production, crop, and soil quality. Many developed countries are using microbes as an alternative to chemical fertilizers. Microbes play a pivotal role in soil fertility through the nutrient cycling process (Patil and Solanki, 2016; Kumari et al., 2020). Different kinds of microbes associated with soil and plants are involved in many fundamental processes, such as biological nitrogen fixation, biotic and abiotic stress regulation, and plant growth promotion (Verma et al., 2020; Mandal et al., 2023). Microbial diversity and function are regulated through the host type, age, ecosystem, climatic condition, and geographical location. The Research Topic “*Emerging Frontiers of Microbial Functions in Sustainable Agriculture*” is in the “Microbe and Virus Interactions with Plants” section in the journal Frontiers in Microbiology. We present a summary of 12 published original research papers.

## Plant microbiome diversity and function

Microbial symbiosis helps plants draw minerals, which boost their growth and defense, through chelation and mineralization. These potential microbiomes can be utilized as bioinoculants to sustain crop production. The structures of plant-associated communities are strongly impacted by soil texture, geography, and agrochemicals, including mineral fertilizers, intercropping systems, developmental stages, and crop rotation (Habig and Swanepoel, 2015; Galazka et al., 2018; Dastogeer et al., 2020; Mandal et al., 2020; Solanki et al., 2020). Kim et al. discussed the decrease of N-cycling communities, such as nifH, archaeal amoA, and nirS, and the increase of bacterial amoA with N fertilization. The report concludes that soil acidification and high nutrient availability disrupt soil N-cycling communities in the cover cropping of corn monocultures. However, cover cropping has a limited impact after 2 years, and long-term applications may cause significant modifications in the microbial communities. On the other hand, Zheng et al. emphasized the importance of tree ages and soil texture in bacterial diversity and composition. *Proteobacteria, Acidobacteria*, and *Actinobacteria* were the dominant bacterial taxa in pomelo tree soil. Soil properties, such as pH and phosphorus availability, play important roles in shifts in bacterial communities. Bacterial genera *Sinomonas* and *Streptacidiphilus* were found to be unique in red soil, while *Actinoallomurus* and *Microbacterium* were found in paddy soil. The microbial co-occurrence network showed that old trees (20 and 30 years) have more complex networks and are more stable than young trees. Li et al. showed that phytase-producing *Pseudomonas* spp. is predominantly found in the alpine grassland of the Qinghai-Tibetan Plateau. These bacteria can promote the growth of *Lolium perenne* L and show multiple plant growth-promoting traits, such as P solubilization, plant production, nitrogen fixation, 1-aminocyclopropane-1-carboxylic acid (ACC) deaminase activity, and antimicrobial activity. Nong et al. described *Burkholderia* sp. strain GXS16, a diazotrophic bacteria colonization response in sugarcane roots. Bacterial colonization enhances antioxidants, such as H_2_O_2_ and malondialdehyde. Differentially expressed genes linked to ethylene pathways were much more highly expressed than those linked to abscisic acid and gibberellin.

## Microbes and agricultural practices

Plant rhizosphere and endosphere-associated microbes are strongly involved in nutrition transport and plant growth development (Solanki et al., 2019). Organic manure positively influences microbial functions and improves soil nutrient availability and uptake by plants (Alori et al., 2017; Jiao et al., 2019). Zhao et al. revealed that cattle manure improves oat plant root length and surface. Bacterial genera *Pseudoxanthomonas, Pseudomonas*, and *Sphingomonas*, and the fungal phylum Ascomycota, were positively related to oat biomass and nutrient accumulation during cattle manure application. Results revealed that Basidiomycota is more abundant in cattle manure deposition treatment than the control. Moreover, cattle manure disrupts the growth of pathotrophs, such as the fungal genera *Alternaria* and *Fusarium*, and encourages the development of saprotrophic and symbiotrophic fungi.

Soil health restoration through different kinds of microbes is an ecological process (Solanki et al., 2019, 2021), and soil health monitoring through microbial activity and response plays an essential role in all restoration processes. Bhaduri et al.. discussed the major strategies that can help restore and maintain ecosystem stability. Various bio-indicators, such as microbial biomass, enzymes, genetic markers, metabolites, and microbial communities, could be used to identify soil health in the presence of different pollutant-contaminated soil samples. Next, Choudhary et al. reported that soil properties and fungal communities play a significant role in climate-smart agricultural (CSA) practices. The fungal taxon Ascomycota was found to be abundant in rice-based CSA scenarios. Additionally, higher levels of soil organic carbon and nitrogen were found in CSA scenarios, improving crop yield.

## Microbes and plants: Biotic and abiotic stresses

Biotic and abiotic factors are considerable limitations in sustainable agriculture production. In recent years, drought stress has become a major issue for agriculture sectors in developing countries (Yandigeri et al., 2012; Wang et al., 2018). Morales-Quintana et al. showed that the fungal endophytes *Penicillium brevicompactum* and *P. chrysogenum*, isolated from Antarctic vascular plants, provoke drought stress regulation in strawberry plants. These endophytes enhance photosynthetic activity, antioxidants, and proline content and reduce lipid peroxidation, which helps plants regulate drought stress. These symbiotic fungi can also be used as an eco-friendly strategy to cope with drought in other crops. Palmieri et al. revealed that patulin biosynthesis by *Penicillium expansum* strongly correlates with extracellular pH in wounded apples. The pH modulation by *Papiliotrema terrestris* LS28 is vital for reducing the amount of patulin. Jia et al. delved into the microbial diversity associated with healthy and wilted *Paeonia suffruticosa* rhizosphere soil. Fungal genera *Fusarium, Cylindrocarpon*, and *Neocosmospora* were directly associated with plant yield reduction and disease incidence. Bacterial and fungal networks were more complex in diseased plants than in healthy ones. The bacterial network significantly impacted the diseased plants that provide a comfortable environment in which the fungal group can grow efficiently. Dastogeer et al. showed that the microbiomes of leaf and grain tissues are altered significantly at the *Magnaporthe oryzae* infection site. The bacterial genus *Rhizobium* increased, whereas the fungal genera *Tylospora, Clohesyomyces*, and *Penicillium* declined in the symptomatic leaf and grain tissues. The microbial network identified several direct interactions between *Magnaporthe oryzae* and other microbes. A higher percentage of soil bacteria was tracked from healthy root samples.

## Fruit-associated microbiome

Microbial communities are associated with fruit surfaces and internal tissues and are impacted by the host's age, evolution, and diversity. The fruit carposphere harbors a wide diversity of microbes (Droby and Wisniewski, 2018). In this regard, Zhimo et al. identified 15 bacterial and 35 fungal core taxa that are abundant at different stages of the apple carposphere of three cultivars that grow in the same environmental conditions. This study represents the strong microbial cross-domain associations, uncovers potential microbe-microbe correlations in the apple carposphere and provides essential information regarding microbial recruitment in the fruit carposphere and its influence over time.

## Conclusion

The non-judicial use of agrochemicals, including fertilizers, and mismanagement of natural soil and water resources greatly impact soil microbial community function, which may result in barren or unproductive soil in the long term. Moreover, soil degradation through the depletion of soil carbon is a critical factor for judging soil carrying capacity and its future utility. In this regard, it is highly pertinent that plant rhizosphere microbes and their symbiotic associations or endophytes play an optimistic candidature, and that their proper use sustains the utility of the soil in the long term.

## Perspectives

- Microbial resources and their significance for maintaining soil ecosystems has been highly recognized in recent decades. However, microbial performance varies greatly depending on the various biotic and abiotic factors that are directly linked with the agroecological conditions.
- Nutrient cycling, restoration of pollutant-contaminated soil, and protection of soil and plant diversity are only possible by the virtue of symbiotic association among microbes in plants and soil.
- Soil health represents the accumulation of healthy soil biota or biodiversity over a period of time or through the proper management of soil biotic components.
- Bridging multiple technologies to better understand the microbial relationships in plant growth and soil productivity. Here, several molecular approaches are applied to extract the in-sight information. However, research and obstacles run parallel with each other, and advanced technologies may help unlock the secret information in the future.

## Author contributions

All authors listed have made a substantial, direct, and intellectual contribution to the work and approved it for publication.
